# Supplementary material for: Regulation of the transcription factor CdnL promotes adaptation to nutrient stress in Caulobacter
Source: bioRxiv. 2023 Dec 21:2023.12.20.572625. Preprint. [Version 1] doi: 10.1101/2023.12.20.572625 (PMC10769358; doi:10.1101/2023.12.20.572625)
Supplement: Supplement 1 [file media-1.pdf]

Supplemental Table S1: Half-life values

Strain/condition	Half-life (min)
WT (EG865) -C	15 ± 2
Δ*spoT* (EG1139) -C	> 500
CdnLDD (EG2530) -C	> 500
SpoTY323A (EG2221) -C	> 500
WT (EG865) -N	18 ± 1
Δ*spoT* (EG1139) -N	60 ± 20
CdnLDD (EG2530) -N	87 ± 19
WT (EG865) -P	> 500
Δ*spoT* (EG1139) -P	> 500
CdnLDD (EG2530) -P	> 500
RelA' (EG1799)	103 ± 16
RelA'-dead (EG1800)	> 500
Δ*cdnL* P_{xyl}-*cdnL* (EG3190) -xyl, -gluc	13 ± 4
Δ*spoT* Δ*cdnL* P_{xyl}-*cdnL* (EG3193) -xyl, -gluc	139 ± 21
SpoTY323A Δ*cdnL* P_{xyl}-*cdnL* (EG3194) -xyl, -gluc	334 ± 288
Δ*cdnL* P_{xyl}-*cdnL* (EG3190) -xyl	27 ± 2
Δ*spoT* Δ*cdnL* P_{xyl}-*cdnL* (EG3193) -xyl	70 ± 12
SpoTY323A Δ*cdnL* P_{xyl}-*cdnL* (EG3194) -xyl	75 ± 8
P_{ruv}-GFP-AA (EG3898)	14 ± 3
Δ*spoT* P_{ruv}-GFP-AA (EG3899)	68 ± 25
WT (EG865)	32 ± 7
CdnLV39A (EG3295)	15 ± 1
CdnLP54A (EG3297)	18 ± 3

Error indicates +/- 1 SD of 3 biological replicates

Supplemental Table S2: Outgrowth and doubling times for Figure 3

	Strain	Approx. time to OD = 0.1 (hr)	t_d (hr)
Pre-starve	WT (EG865)	4.2	2.1 ± 0.06
	CdnLDD (EG2530)	4.5	2.2 ± 0.02
	RNAP-1 (EG2652)	4.5	2.2 ± 0.02
	Δ*dksA* (EG2756)	4.0	2.1 ± 0.15
	RNAP-1/Δ*dksA* (EG2776)	4.8	2.3 ± 0.02
	CdnLDD/RNAP-1/Δ*dksA* (EG2861)	5.5	2.5 ± 0.24
Post-starve	WT (EG865)	8.3	1.9 ± 0.14
	CdnLDD (EG2530)	9.3	2.1 ± 0.02
	RNAP-1 (EG2652)	9.1	2.2 ± 0.09
	Δ*dksA* (EG2756)	10.3	2.6 ± 0.19
	RNAP-1/Δ*dksA* (EG2776)	10.5	2.9 ± 0.52
	CdnLDD/RNAP-1/Δ*dksA* (EG2861)	11.6	2.7 ± 0.05

Error indicates +/- 1 SD of 3 biological replicates

Supplemental Table S3: Plasmids and Strains used in this study

Dataset S1: ChIP-seq data of WT CdnL and CdnLDD in M2G and 60 minutes in M2

Dataset S2: RNA-seq comparing WT (EG865) to CdnLDD (EG2530) at 0 minutes of starvation
Includes all quantified genes, genes > 2-fold differentially regulated (p < 0.05 and FDR < 0.05), and DAVID analyses

Dataset S3: RNA-seq comparing WT (EG865) to CdnLDD (EG2530) at 60 minutes of starvation
Includes all quantified genes, genes > 2-fold differentially regulated (p < 0.05 and FDR < 0.05), and DAVID analyses

Dataset S4: RNA-seq data comparing WT (EG865) 60 minutes starved to 24 hours starved
Includes all quantified genes and genes > 2-fold differentially regulated (p < 0.05 and FDR < 0.05)

Dataset S5: RNA-seq comparing WT (EG865) to CdnLDD (EG2530) at 24 hours of starvation

Includes all quantified genes, genes > 2-fold differentially regulated (p < 0.05 and FDR < 0.05), and DAVID analyses

Dataset S6: RNA-seq comparing WT (EG865) to CdnLDD (EG2530) at 60 minutes of recovery after glucose addition

Includes all quantified genes, genes > 2-fold differentially regulated (p < 0.05 and FDR < 0.05), and DAVID analyses
