# Supplementary material for: Regulation of the transcription factor CdnL promotes adaptation to nutrient stress in Caulobacter
Source: bioRxiv. 2023 Dec 21:2023.12.20.572625. Preprint. [Version 1] doi: 10.1101/2023.12.20.572625 (PMC10769358; doi:10.1101/2023.12.20.572625)
Supplement: 9 [file NIHPP2023.12.20.572625v1-supplement-9.pdf]

bioRxiv preprint doi: https://doi.org/10.1101/2023.12.20.572625; this version posted December 21, 2023. The copyright holder for this preprint (which was not certified by peer review) is the author/funder, who has granted bioRxiv a license to display the preprint in perpetuity. It is made available under aCC-BY 4.0 International license.

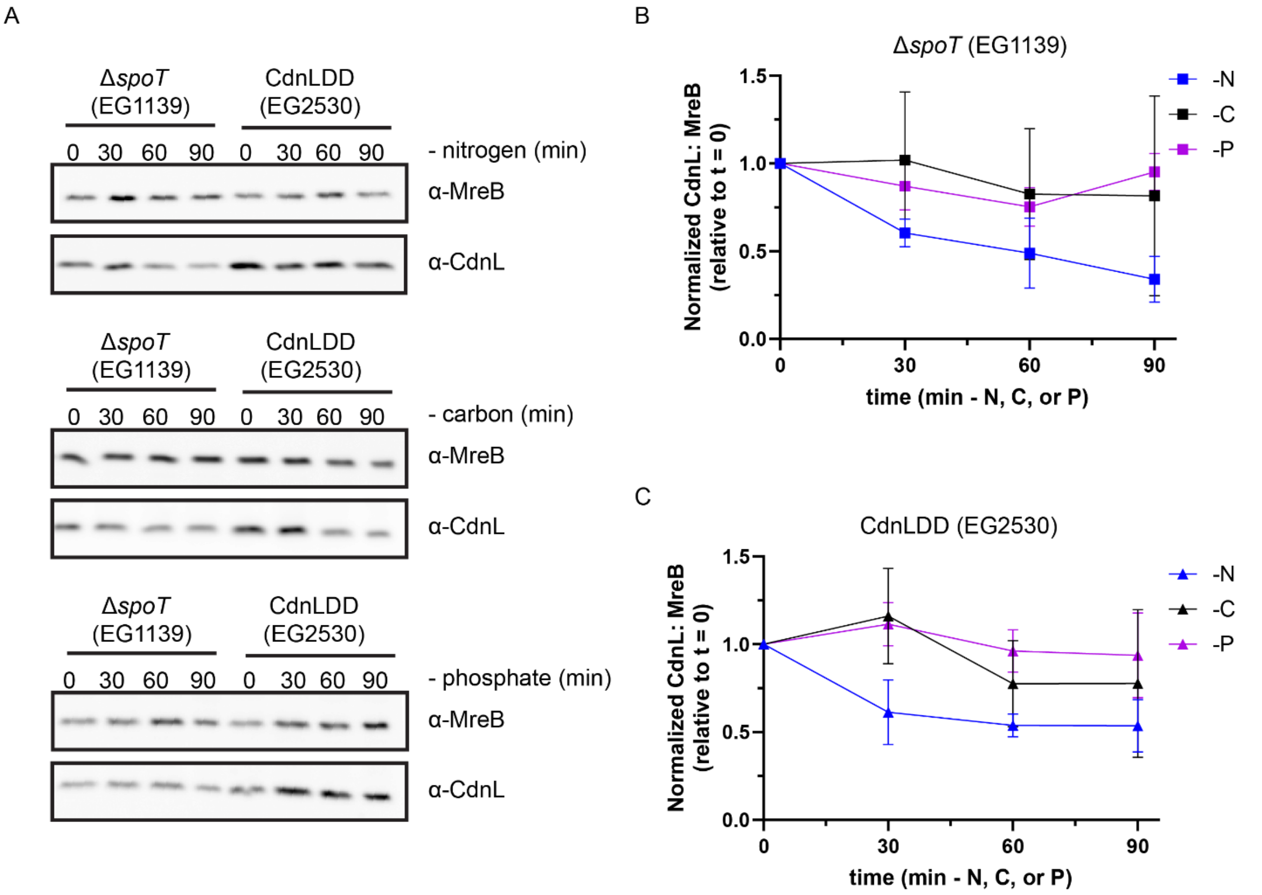

Supplemental Figure S1: CdnL levels are stabilized in ΔspoT and CdnLDD

A. Representative western blot of CdnL during 90 minutes of nitrogen, carbon, and phosphate starvation in Δ*spoT* (EG1139) and CdnLDD (EG2530). Protein samples were taken every 30 minutes. MreB was used as a loading control.

B. Densitometry of CdnL levels (normalized to MreB) relative to t = 0 during nitrogen (-N), carbon (-C), and phosphate (-P) starvations in Δ*spoT* (EG1139) from western blots as performed in A. Error bars represent +/- 1 SD of 3 biological replicates.

C. Densitometry of CdnL levels (normalized to MreB) relative to t = 0 during nitrogen (-N), carbon (-C), and phosphate (-P) starvations in CdnLDD (EG2530) from

bioRxiv preprint doi: https://doi.org/10.1101/2023.12.20.572625; this version posted December 21, 2023. The copyright holder for this preprint (which was not certified by peer review) is the author/funder, who has granted bioRxiv a license to display the preprint in perpetuity. It is made available under aCC-BY 4.0 International license.

western blots as performed in A. Error bars represent +/- 1 SD of 3 biological replicates.

bioRxiv preprint doi: https://doi.org/10.1101/2023.12.20.572625; this version posted December 21, 2023. The copyright holder for this preprint (which was not certified by peer review) is the author/funder, who has granted bioRxiv a license to display the preprint in perpetuity. It is made available under aCC-BY 4.0 International license.

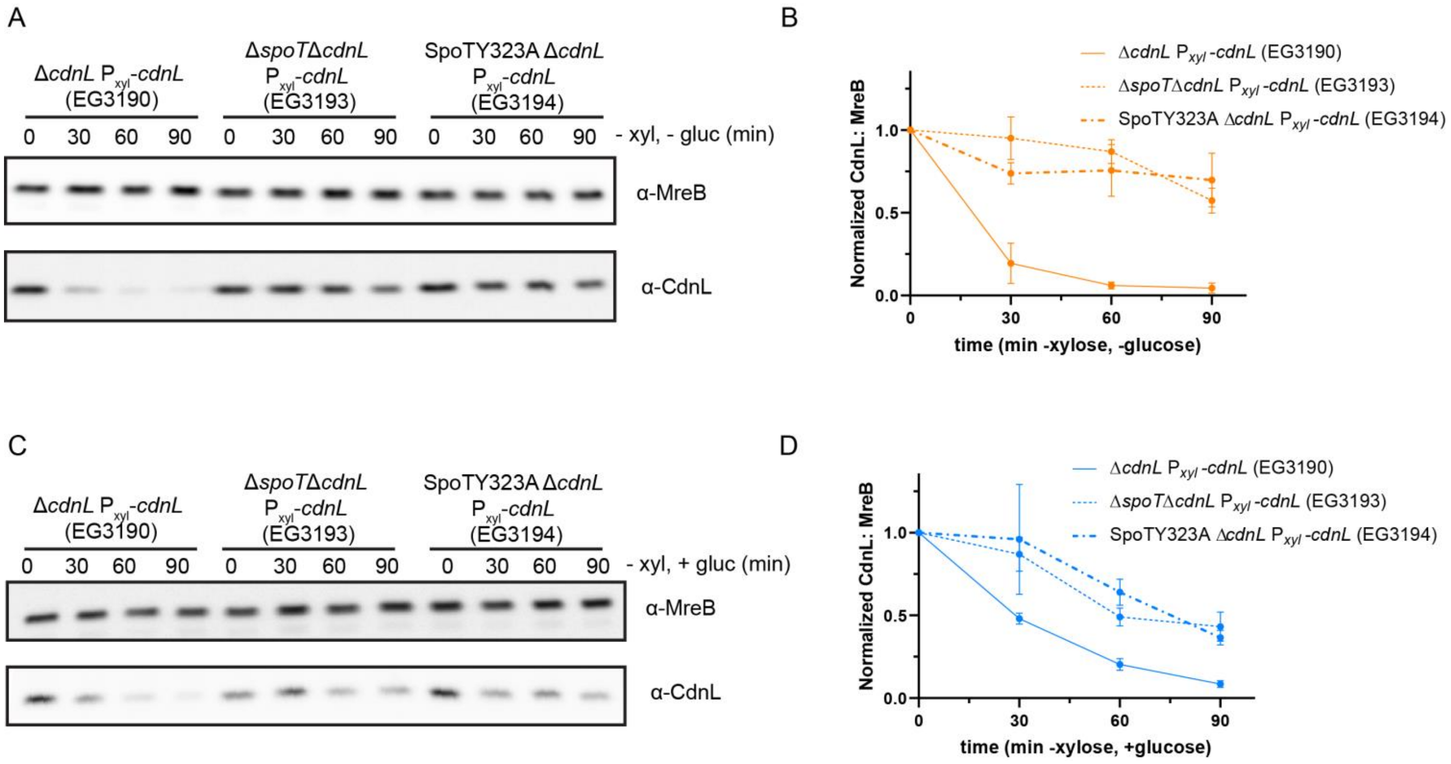

Supplemental Figure S2: Transcriptional regulation of *cdnL* is not sufficient to control CdnL levels

A. Representative western blot of CdnL during 90 minutes of glucose (gluc) starvation with xylose (xyl) depletion of *cdnL* in a Δ*cdnL,* Δ*spoT*Δ*cdnL*, or SpoTY323AΔ*cdnL* background. Protein samples were taken every 30 minutes. MreB was used as a loading control.

B. Densitometry of CdnL levels (normalized to MreB) relative to t = 0 from western blots as performed in A. Error bars represent +/- 1 SD of 3 biological replicates.

C. Representative western blot of CdnL during xylose (xyl) depletion of *cdnL* in a Δ*cdnL,* Δ*spoT*Δ*cdnL*, or SpoTY323AΔ*cdnL* background. Protein samples were taken every 30 minutes. MreB was used as a loading control.

bioRxiv preprint doi: https://doi.org/10.1101/2023.12.20.572625; this version posted December 21, 2023. The copyright holder for this preprint (which was not certified by peer review) is the author/funder, who has granted bioRxiv a license to display the preprint in perpetuity. It is made available under aCC-BY 4.0 International license.

D. Densitometry of CdnL levels (normalized to MreB) relative to t = 0 from western blots as performed in C. Error bars represent +/- 1 SD of 3 biological replicates.

bioRxiv preprint doi: https://doi.org/10.1101/2023.12.20.572625; this version posted December 21, 2023. The copyright holder for this preprint (which was not certified by peer review) is the author/funder, who has granted bioRxiv a license to display the preprint in perpetuity. It is made available under aCC-BY 4.0 International license.

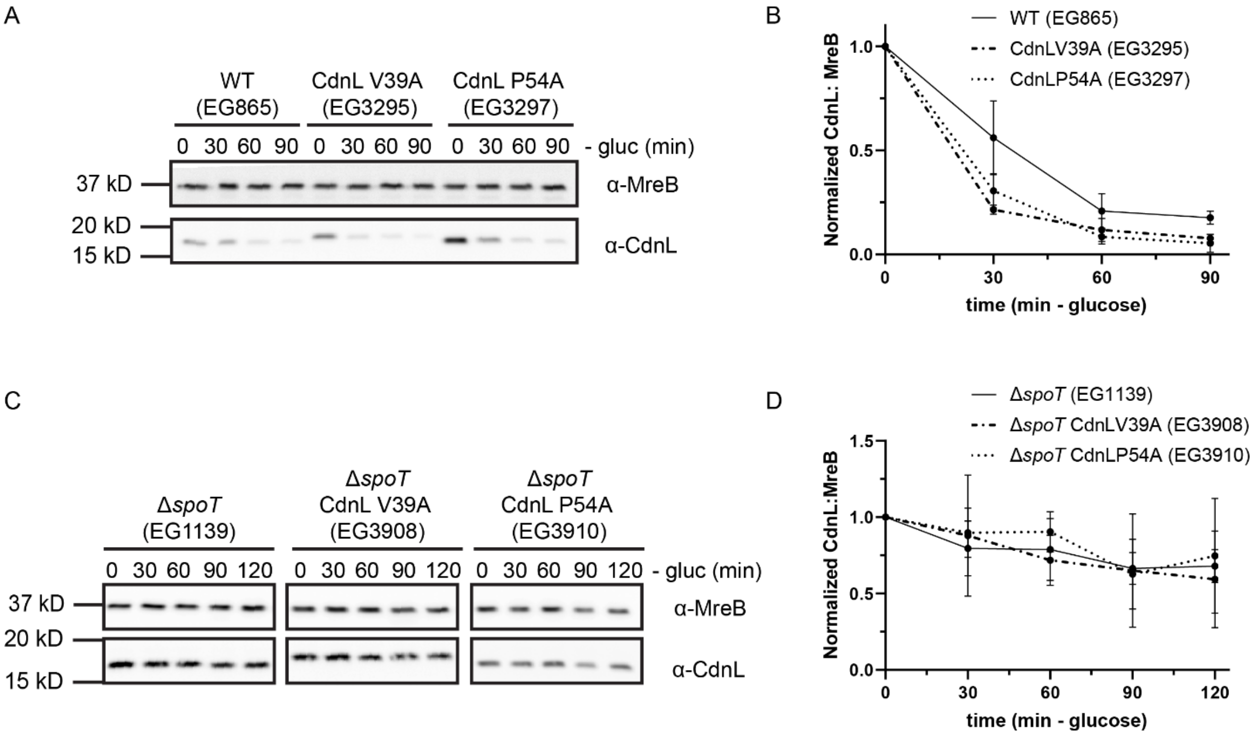

Supplemental Figure S3: CdnL mutants with a reduced interaction with RNAP are cleared more rapidly than WT CdnL

A. Representative western blot of WT CdnL, CdnL V39A, or CdnL P54A during 90 minutes of glucose (gluc) starvation. Protein samples were taken every 30 minutes. MreB was used as a loading control.

B. Densitometry of WT CdnL, CdnL V39A, or CdnL P54A levels (normalized to MreB) relative to t = 0 from western blots as performed in A. Error bars represent +/- 1 SD of 3 biological replicates.

C. Representative western blot of WT CdnL, CdnL V39A, or CdnL P54A in a Δ*spoT* background during 90 minutes of glucose (gluc) starvation. Protein samples were taken every 30 minutes. MreB was used as a loading control.

bioRxiv preprint doi: https://doi.org/10.1101/2023.12.20.572625; this version posted December 21, 2023. The copyright holder for this preprint (which was not certified by peer review) is the author/funder, who has granted bioRxiv a license to display the preprint in perpetuity. It is made available under aCC-BY 4.0 International license.

D. Densitometry of GFP-AA levels (normalized to SpmX) relative to t = 0 from western blots as performed in A. Error bars represent +/- 1 SD of 3 biological replicates.

bioRxiv preprint doi: https://doi.org/10.1101/2023.12.20.572625; this version posted December 21, 2023. The copyright holder for this preprint (which was not certified by peer review) is the author/funder, who has granted bioRxiv a license to display the preprint in perpetuity. It is made available under aCC-BY 4.0 International license.

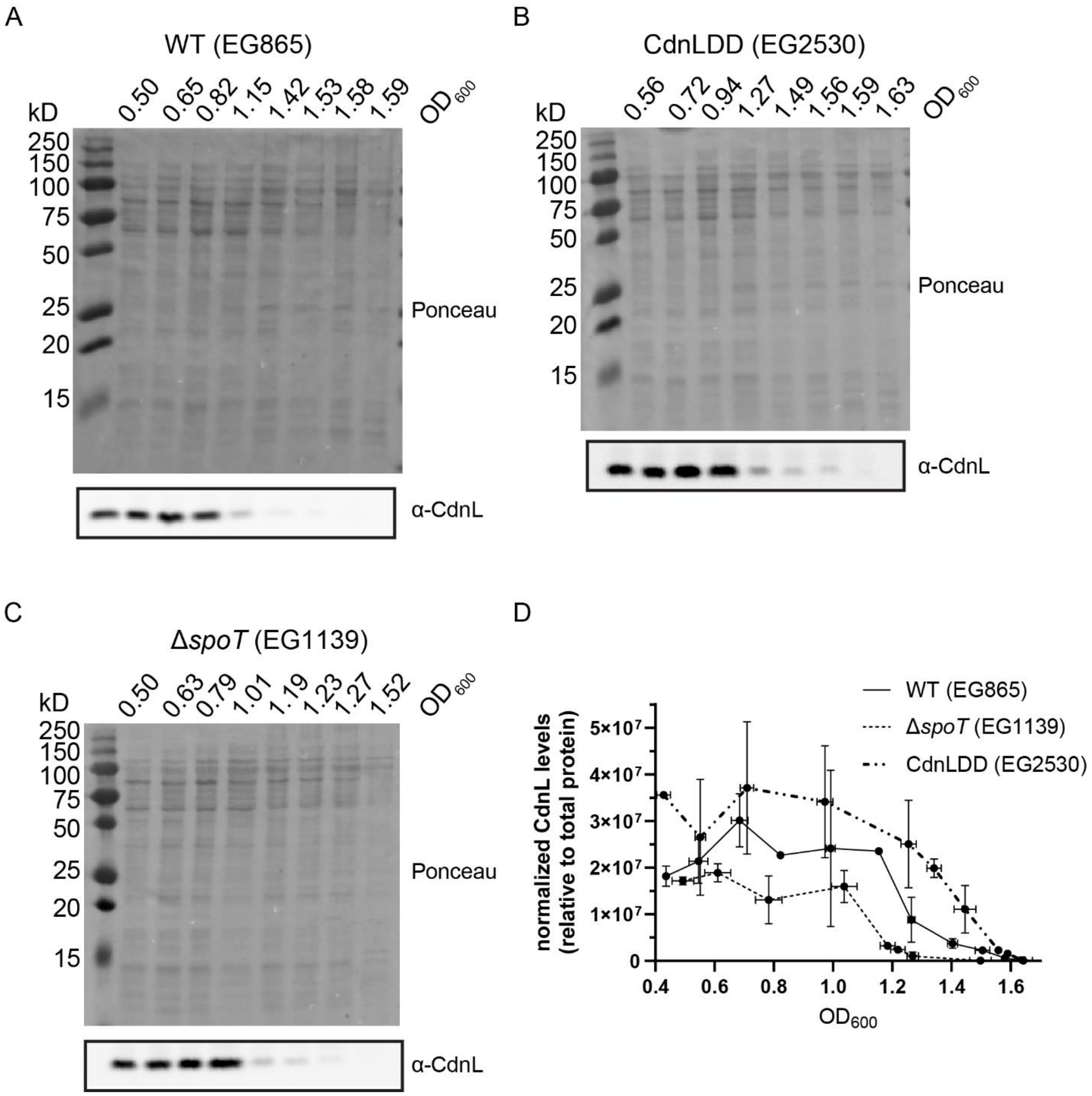

Supplemental Figure S4: CdnL clearance during stationary phase is SpoT- and ClpXP-independent

A. Representative Ponceau stain and western blot for CdnL in WT (EG865). Samples were taken once OD_{600} = 0.4 – 0.6 for t = 0, and then at t = 1, 2, 4, 6, 7, 8, and 25 hours later. OD_{600} was recorded at each timepoint.

bioRxiv preprint doi: https://doi.org/10.1101/2023.12.20.572625; this version posted December 21, 2023. The copyright holder for this preprint (which was not certified by peer review) is the author/funder, who has granted bioRxiv a license to display the preprint in perpetuity. It is made available under aCC-BY 4.0 International license.

B. Representative Ponceau stain and western blot for CdnL in Δ*spoT* (EG1139). Samples were taken once OD_{600} = 0.4 – 0.6 for t = 0, and then at t = 1, 2, 4, 6, 7, 8, and 25 hours later. OD_{600} was recorded at each timepoint.

C. Representative Ponceau stain and western blot for CdnL in CdnLDD (EG2530). Samples were taken once OD_{600} = 0.4 – 0.6 for t = 0, and then at t = 1, 2, 4, 6, 7, 8, and 25 hours later. OD_{600} was recorded at each timepoint.

D. Densitometry analysis of CdnL/CdnLDD (normalized to total protein) for WT, Δ*spoT,* or CdnLDD strains from OD_{600} ~ 0.4 – 1.6 using Ponceau-stained membranes and western blots as performed in A - C. CdnL levels for OD_{600} values within 0.1 units were averaged. X and Y error bars represent +/- 1 SD of 1 – 3 biological replicates per point.

bioRxiv preprint doi: https://doi.org/10.1101/2023.12.20.572625; this version posted December 21, 2023. The copyright holder for this preprint (which was not certified by peer review) is the author/funder, who has granted bioRxiv a license to display the preprint in perpetuity. It is made available under aCC-BY 4.0 International license.

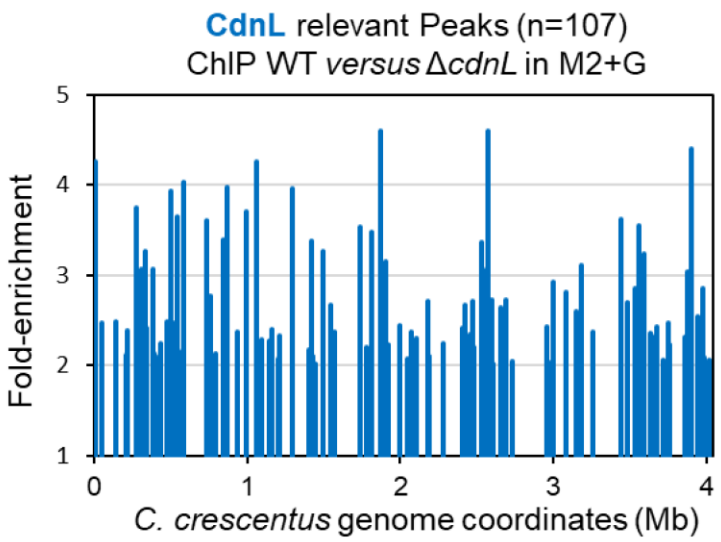

Supplemental Figure S5: CdnL relevant peaks identified by ChIP-seq

107 peaks identified by ChIP-seq as having a > 2-fold enrichment compared to the Δ*cdnL* (EG1898) control in M2G. These peaks were selected for further analyses in comparing WT (EG865) to CdnLDD (EG2530) in M2G and M2.

bioRxiv preprint doi: https://doi.org/10.1101/2023.12.20.572625; this version posted December 21, 2023. The copyright holder for this preprint (which was not certified by peer review) is the author/funder, who has granted bioRxiv a license to display the preprint in perpetuity. It is made available under aCC-BY 4.0 International license.

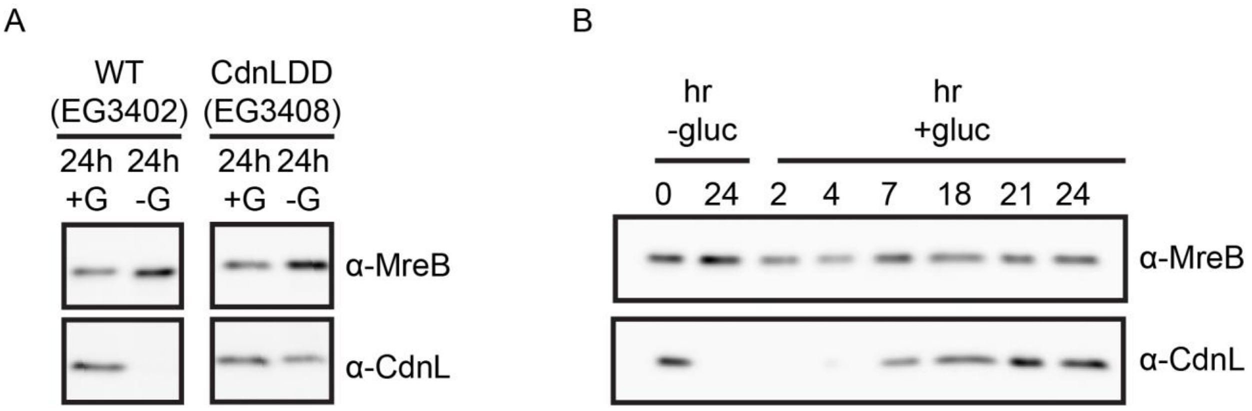

Supplemental Figure S6: CdnLDD is stable after 24 hours of starvation

A. Representative western blot of WT (EG3402) and CdnLDD (EG3408) before (24h +G) and after (24h -G) 24 hours of starvation. MreB was used as a loading control.

B. Representative western blot of CdnL levels in WT (EG3402) after 24 hours of starvation and at indicated timepoints following the addition of glucose. MreB was used as a loading control.
